# Structural basis for inhibition of SpyCas9 by the anti-CRISPR protein AcrIIA26

**DOI:** 10.1042/BCJ20250364

**Published:** 2026-02-06

**Authors:** Iris Zheng, Brian Learn, Scott Bailey

**Affiliations:** 1 Department of Biochemistry and Molecular Biology, Johns Hopkins University, Bloomberg School of Public Health, Baltimore, MD 21205, U.S.A.; 2 Department of Biophysics and Biophysical Chemistry, Johns Hopkins University, School of Medicine, Baltimore, MD 21205, U.S.A.

**Keywords:** Acr, Cas9, CRISPR

## Abstract

CRISPR–Cas9 systems provide adaptive immunity in prokaryotes by targeting and cleaving invading phage DNA. In response, phages have evolved anti-CRISPR (Acr) proteins to inhibit Cas9 and evade this immune response. AcrIIA26 is a type II-A anti-CRISPR protein that inhibits *Streptococcus pyogenes* Cas9 (SpyCas9) DNA binding, but its molecular mechanism remains unclear. Here, we determined the 3.0 Å resolution cryo-EM structure of AcrIIA26 in complex with SpyCas9–single-guide RNA, revealing a dual inhibition mechanism. AcrIIA26 adopts a novel fold comprising a central β-sheet flanked by two α-helical bundles. The 5-helix bundle, which features a negatively charged surface whose shape mimics duplex DNA, occupies the same position as the protospacer adjacent motif (PAM) duplex in target-bound Cas9. This directly blocks PAM recognition by burying critical residues R1333 and R1335 in the PAM-interacting domain. Mutagenesis confirmed that residues E49 and D50 in AcrIIA26 are essential for this interaction. Simultaneously, the 4-helix bundle binds the Cas9 REC lobe and sterically prevents the conformational changes required for Cas9 activation, with mutation of AcrIIA26 F121 completely eliminating inhibitory activity. Structural comparisons reveal that despite diverse folds, multiple anti-CRISPRs convergently evolved to block PAM recognition, highlighting this as a critical vulnerability in Cas9 function. Our findings provide mechanistic insights into AcrIIA26 inhibition and offer a foundation for engineering improved Cas9 off-switches for genome editing applications.

## Introduction

CRISPR–Cas (clustered regularly interspaced short palindromic repeats–CRISPR-associated proteins) systems are prokaryotic adaptive immune systems that target and eliminate invading phage and mobile genetic elements [1]. Infection is neutralized in three phases: adaptation, biogenesis, and interference. During the adaptation, the invasion of foreign nucleic acid triggers the integration of foreign DNA into the CRISPR array within the prokaryotic genome [2–4]. In biogenesis, the Cas proteins are expressed, and the CRISPR array is transcribed and processed into mature CRISPR RNA (crRNA) [5]. During interference, the crRNA guides a Cas effector complex to detect and degrade the foreign genetic material [6]. In DNA targeting systems, activity not only requires base pairing between the crRNA and its complementary sequence in the DNA target (the protospacer) but also requires a protospacer adjacent motif (PAM) [7]. CRISPR arrays lack PAMs, ensuring that the host genome is not targeted, thus providing a mechanism to distinguish self from non-self DNA [7,8].

**Figure 1:**
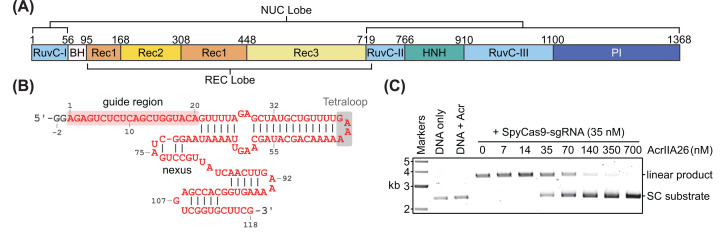
Cas9 and its inhibition by AcrIIA26 (**A**) Domain organization of *Streptococcus pyogenes* Cas9 (SpyCas9). (**B**) Schematic of the sgRNA used in this study. The guide region targets the TRAC gene. (**C**) SpyCas9 cleavage assay in the presence of increasing concentrations of AcrIIA26. SC, supercoiled; kb, kilobase pairs.

CRISPR–Cas systems are highly diverse and can be classified into 2 classes, 7 types, and over 30 subtypes [9–12]. Class 1 systems (types I, III, IV, and VII) feature multi-subunit effector complexes, while class 2 systems (types II, V, and VI) feature an effector composed of a single Cas protein. Cas9, the type II effector, cleaves both strands of the DNA target to produce double-stranded breaks (DSBs). The ability to program Cas9 to introduce DSB at specific sites defined by an RNA sequence has led to its widespread adoption as a versatile gene editing platform [13]. Cas9 activity also requires a *trans*-activating crRNA (tracrRNA) that mediates both crRNA loading and subsequent DNA binding and cleavage [14,15]. For simplicity, in most Cas9-based applications, the crRNA and tracrRNA are joined into a single-guide RNA (sgRNA) [15]. The Cas9 protein has multiple domains (Figure 1A) that are arranged into two lobes [16,17]. The REC lobe, containing the REC1, REC2, and REC3 domains, is primarily responsible for binding RNA and facilitates target strand binding [16,18]. The NUC lobe contains the PAM-interacting (PI) domain, which mediates initial DNA binding and PAM recognition [19], and the two nuclease domains responsible for cleaving the target DNA: the HNH domain that cleaves the target strand and the RuvC domain that cleaves the non-target strand [15–17,20]. The REC and NUC lobes are connected by a long arginine-rich bridge helix (BH). The BH is essential for Cas9 activity and makes direct and indirect contacts with the sgRNA and target DNA [16,17,21,22].

In response to the arms race against bacteria, phages have developed small inhibitory proteins called anti-CRISPRs (Acrs) to escape prokaryotic adaptive immunity [23]. Currently, there are at least 46 distinct families of Acrs with little or no sequence or structural similarity between them [23–25]. Acrs are named after the CRISPR–Cas subtype they inhibit (e.g. II-A) and are numbered sequentially as they are discovered [26]. Acrs that inhibit type II-A systems do so by one of three distinct mechanisms: (1) they prevent Cas9–sgRNA complex formation, either by preventing sgRNA from being loaded onto Cas9 (e.g. AcrIIA6 and AcrIIA17) or by triggering Cas9 degradation (e.g. AcrIIA1) [27–32]; (2) they prevent Cas9 binding to its DNA target (e.g. AcrIIA2, AcrIIA4, and AcrIIA26) [33–38]; or (3) they prevent Cas9 target cleavage (e.g. AcrIIA5) [32,37,39,40].

The inhibition by Acrs can also be exploited for various applications. One important application of Acr is as an ‘off-switch’ for CRISPR–Cas9 editing. Despite the vast potential of CRISPR–Cas9 as a genome editing tool, its off-target effects remain a major obstacle to research and clinical applications. Off-target effects occur when Cas9 cleaves a DNA sequence with high but imperfect complementarity with the sgRNA, resulting in unintended editing events. The risk of off-target effects increases the longer Cas9 remains in the edited cell [41]. Acrs, as natural inhibitors of the CRISPR–Cas systems, are perfect Cas9 controllers due to their high specificity [23]. Indeed, timed delivery of an Acr into human cells during Cas9 editing reduced off-target effects without sacrificing editing efficiency [36]. Another application of Acrs is as an antibiotic. The overuse of conventional antibiotics causes bacteria to acquire antibiotic resistance, reducing their efficiency [42,43]. Since they are capable of inhibiting the bacterial adaptive immune system, Acrs offer a viable alternative for treating bacterial infection [44].

While Acrs hold significant potential for research and clinical applications, many lack the structural characterization that would greatly aid in realizing their potential. One such Acr is AcrIIA26, a type II-A anti-CRISPR protein encoded by *Streptococcus* phage that inhibits DNA binding by SpyCas9 [37]. Here, we determined the 3.0 Å resolution cryogenic electron microscopy (cryo-EM) structure of AcrIIA26 in complex with SpyCas9 loaded with sgRNA. Our structural and biochemical analyses demonstrate that AcrIIA26 employs a dual inhibition strategy, preventing both DNA binding and Cas9 activation.

## Results

### Structure of AcrIIA26 bound to SpyCas9–sgRNA

To elucidate the structural mechanism by which AcrIIA26 inhibits SpyCas9 activity, we set out to determine the structure of the complex using single-particle cryo-EM. Before beginning cryo-EM studies, we tested AcrIIA26 inhibition of SpyCas9 DNA cleavage using our purified proteins. To simplify complex formation, the crRNA and tracrRNA were fused with a GAAA tetra-loop to produce an sgRNA (Figure 1B) [15,21]. SpyCas9 preloaded with sgRNA was incubated with increasing concentrations of AcrIIA26 and then introduced to a supercoiled plasmid containing a protospacer and NGG-PAM (the PAM for SpyCas9). Under our assay conditions and in the absence of AcrIIA26, SpyCas9–sgRNA completely cleaves the target sequence, linearizing the supercoiled plasmid (Figure 1C). In the presence of AcrIIA26, inhibition gradually increases as the concentration of AcrIIA26 increases, indicating that AcrIIA26 reduces SpyCas9 cleavage in a concentration-dependent manner in our assay (Figure 1C).

**Figure 2:**
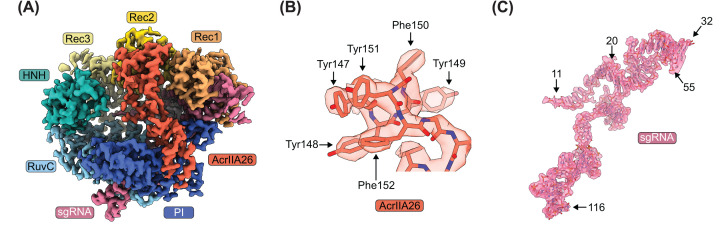
Cryo-EM structure of the Cas9–sgRNA–AcrIIA26 complex (**A**) Cryo-EM reconstruction of the Cas9–sgRNA–AcrIIA26 complex at 3.0 Å resolution. (**B**) Representative cryo-EM density for AcrIIA26 with refined model superimposed. (**C**) Cryo-EM density for the sgRNA with refined model superimposed.

To determine the structure of the SpyCas9–AcrIIA26 complex, we mixed 4.0 μM SpyCas9–sgRNA with 8.0 μM AcrIIA26 and directly prepared cryo-EM grids from this sample. Using these grids, we determined the structure of SpyCas9–sgRNA–AcrIIA26 to an overall resolution of 3.0 Å (Figure 2A). We observed density for a single AcrIIA26 with clear side-chain features (Figure 2B). Density for the majority of SpyCas9 also displayed clear side-chain features. However, due to its inherent flexibility [16,45], the density describing the HNH domain was much weaker and displayed lower resolution than the rest of the complex. To improve the HNH density, we performed particle subtraction and local refinement. This greatly enhanced the density in this region, improving the local resolution from 4.1 to 3.3 Å (Supplementary Figures S1 and S2). Density describing the nucleotides in the sgRNA was also clearly defined, except for nucleotides 1 to 10 of the guide RNA segment, part of the hairpin containing the tetraloop (nucleotides 33 to 53), and nucleotides 117 and 118 (Figure 2C).

**Figure 3:**
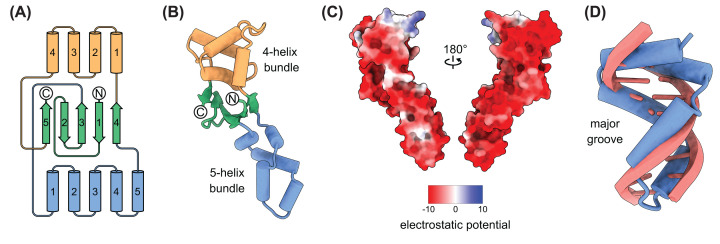
Structure of AcrIIA26 (**A**) Topology diagram of the AcrIIA26 structure. (**B**) Ribbon diagram of the AcrIIA26 structure. (**C**) Electrostatic potential surface of AcrIIA26. (**D**) Overlay of the AcrIIA26 5-helix bundle domain with dsDNA.

AcrIIA26 comprises 183 amino acids forming a five-stranded antiparallel β-sheet flanked by a five α-helical bundle (5-helix bundle) on one side and a four α-helical bundle (4-helix bundle) on the other (Figure 3A). The overall structure resembles a bow where the two α-helical bundles represent the loops that are tied together by the central β-sheet (Figure 3B). No similar structure was found by a search with the DALI server [46], indicating that AcrIIA26 has a novel fold. The overall surface of AcrIIA26 is highly acidic, with a nearly uniform negative charge and a theoretical pI of 4.12 (Figure 3C). Notably, the negatively charged surface of the 5-helix bundle has a shape that closely resembles that of duplex DNA (Figure 3D).

**Figure 4:**
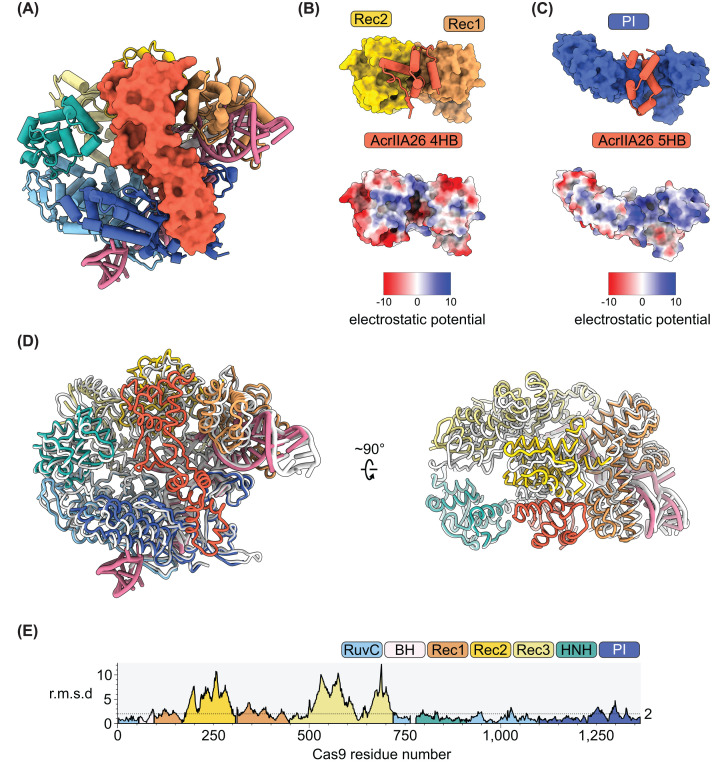
AcrIIA26 binds both lobes of Cas9 and traps Cas9 in an inactive conformation (**A**) The atomic model of SpyCas9–sgRNA–AcrIIA26. Cas9 and the sgRNA are shown in ribbon diagram, and AcrIIA26 is displayed as surface representation. Colors are as in Figure 2A. (**B**) Top: Interaction of the AcrIIA26 4-helix bundle and the Rec1 and Rec2 domains of Cas9. Bottom: Electrostatic potential surfaces of the Rec1 and Rec2 domains of Cas9. (**C**) Top: Interaction of the AcrIIA26 5-helix bundle and the PI domain of Cas9. Bottom: Electrostatic potential surface of the PI domain of Cas9. (**D**) Structural comparison of the Cas9–sgRNA–AcrIIA26 complex (colored as in Figure 2A) and the Cas9–sgRNA complex (white). (**E**) Plot of r.m.s.d per residue of Cas9 between the Cas9–sgRNA–AcrIIA complex and the Cas9–sgRNA complex.

In the AcrIIA26–SpyCas9–sgRNA complex, AcrIIA26 spans across the central channel formed by the REC and NUC lobes, burying 1903 Å^2^ of surface area (Figure 4A). AcrIIA26 directly contacts the REC1 and REC2 domains with its 4-helix bundle (Figure 4B) and the PI domain with its 5-helix bundle (Figure 4C). These interactions are facilitated by the positively charged patches on the surface of these Cas9 domains and the negatively charged surface of AcrIIA26. The central β-sheet acts as a spacer between the two α-helical bundles and does not make direct contact with Cas9 or the sgRNA. No interactions are observed with the nuclease domains, the REC3 domain, the bridge helix, or the sgRNA. Overall the conformation of Cas9 in the complex most closely resembles the conformation of the inactive pre-target bound state (Figure 4D) [47], except that AcrIIA26 binding repositions the REC2 and REC3 domains (Figure 4E).

### AcrIIA26 directly blocks target DNA binding and Cas9 activation

**Figure 5:**
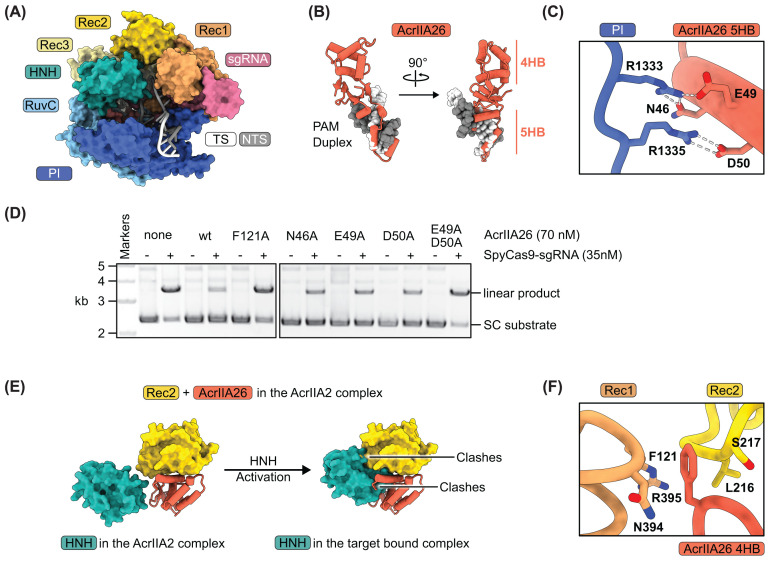
Comparison of the Cas9–sgRNA–AcrIIA26 and Cas9–sgRNA–target DNA complexes (**A**) Atomic model of the Cas9–sgRNA–target DNA complex (PBD ID 4UN3). (**B**) Superimposition of the Cas9–sgRNA–AcrIIA26 complex with the Cas9–sgRNA–target DNA complex. For clarity, only AcrIIA26 (ribbon diagram) and the PAM duplex DNA (surface representation) are shown. (**C**) Close-up view showing how AcrIIA26 interacts with the PAM recognition residues (R1333 and R1335) of SpyCas9. Hydrogen bonds are indicated with dashed white lines. (**D**) Cas9 DNA cleavage assays in the presence of wild-type and mutant AcrIIA26. SC, supercoiled; kb, kilobase pairs. (**E**) Shown on the left are the relative positions of the HNH and Rec2 domains of SpyCas9 along with AcrIIA26 in the Cas9–sgRNA–AcrIIA26 complex. Shown on the right is how movement of the HNH domain into the position it occupies in the Cas9–sgRNA–target DNA complex would clash with the Rec2 domain and AcrIIA26. (**F**) Close-up view showing how AcrIIA26 F121 interacts with the Cas9 Rec1 and Rec2 domains.

Comparison of SpyCas9’s target-bound (Figure 5A) [19] and AcrIIA26-bound structures reveals that the 5-helix bundle of AcrIIA26 occupies the same position as the PAM duplex DNA (Figure 5B) contacting the PI domain in the NUC lobe (Figure 4C). Residues R1333 and R1335 within the PI domain are essential for recognition of the NGG-PAM [19]. Both of these residues are buried in the AcrIIA26-bound structure. AcrIIA26 residues N46 and E49 interact with R1333, while D50 interacts with R1335 (Figure 5C and Supplementary Figure S3A). To characterize the importance of these AcrIIA26 residues, we generated alanine substitution mutations and conducted DNA cleavage assays with each mutant. All three individual mutants (N46A, E49A, and D50A) modestly impaired inhibition by AcrIIA26 (Figure 5D). However, the E49A/D50A double mutant almost completely abolished its activity, resembling the no AcrIIA26 control (Figure 5D). In addition to blocking the PAM interaction, AcrIIA26 binding also buries residues critical for target DNA unwinding. F47 of AcrIIA26 forms van der Waals contacts with S1136 within the PI domain of Cas9 (Supplementary Figure S3B). In the target-bound structure, S1136 hydrogen bonds with the +1 phosphate on the target strand immediately upstream of the PAM (an interaction called the phosphate lock), stabilizing the unwound DNA [19]. Thus, AcrIIA26 binding blocks this stabilization.

The 4-helix bundle of AcrIIA26 sits in the REC lobe, contacting both the REC1 and REC2 domains primarily via charge–charge interactions (Figure 4B). Notably, however, the side chain of F121 from AcrIIA26 is inserted between the REC1 and REC2 domains, making contact with backbone atoms or the C_β_ of Cas9 residues L216, S217, N394, and R395 (Figure 5F and Supplementary Figure S3C). Mutation of F121 to alanine (F121A) abolished inhibition by AcrIIA26 (Figure 5D). DNA cleavage by Cas9 is controlled by a conformational change of the HNH nuclease domain. In the Cas9–sgRNA–AcrIIA26 complex, the 4-helix bundle of AcrIIA26 and the REC2 domain are in a position that the HNH domain occupies in the activated Cas9 state (Figure 5E), thereby preventing activation. Together these data indicate that interaction between the 4-helix bundle and the REC lobe of Cas9 is essential for AcrIIA26 function.

### Comparing the structures of the Cas9 bound to different AcrII proteins

**Figure 6:**
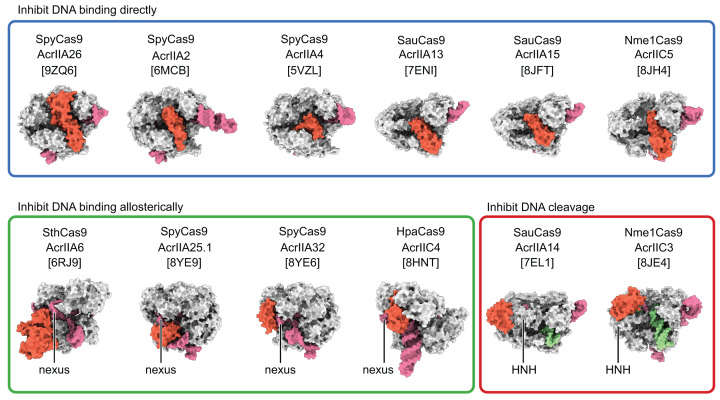
Comparison of Cas9–AcrII complexes Atomic models of Cas9 in complex with AcrII proteins displayed as surface representations. PDB ID codes are shown in square brackets. Cas9 is white, the sgRNA is pink, AcrII proteins are red, and target DNA where applicable is green. All Cas9 structures are in the same orientation except those in the green box (inhibit DNA binding allosterically), which are rotated 180**°** about the *y*-axis. Spy, *Streptococcus pyogenes*; Sau, *Staphylococcus aureus*; Nme1, *Neisseria meningitidis* 1; Sth, *Streptococcus thermophilus*; and Hpa, *Haemophilus parainfluenzae*. Note that in the Nme1Cas9–AcrIIC3 structure, two AcrIIC3 proteins tether two Nme1Cas9 proteins together, but for clarity, only half this complex is shown.

To understand how inhibition of Cas9 by AcrIIA26 compares to inhibition by other Acrs, we aligned the structures of all experimentally determine Cas9–AcrII complexes. To date there are 11 such structures: 8 with Cas9 II-A and 3 with Cas9 II-C (Figure 6). All of the Acrs, regardless of mechanism, have distinct folds (Supplementary Figure S4). AcrIIA14 and AcrIIC3 bind to the HNH domain of Cas9 inhibiting DNA cleavage but not DNA binding (Figure 6) [48,49]. Although the details differ, AcrIIA6, AcrIIA25.1, AcrIIA32, and AcrIIC4 all contact the nexus of the sgRNA (Figure 1B) [50], on the opposite face of Cas9 to the DNA binding site (Figure 6). This binding inhibits Cas9 activity allosterically by preventing conformational changes required for DNA binding [27,51,52]. AcrIIA2, AcrIIA4, AcrIIA13, AcrIIA15, and AcrIIC5 all inhibit PAM recognition by directly blocking the PAM binding pocket of Cas9 in a manner highly reminiscent of AcrIIA26 (Figure 6) [33,34,53,54]. Indeed, in all these structures the residues used by Cas9 to recognize the PAM sequence are specifically contacted by residues within the Acr.

## Discussion

Here, we determined the 3.0 Å resolution cryo-EM structure of AcrIIA26 in complex with SpyCas9–sgRNA (Figure 2A), revealing the molecular mechanism by which this anti-CRISPR protein inhibits SpyCas9 activity. Our structural and biochemical analyses demonstrate that AcrIIA26 employs a dual inhibition strategy: it directly blocks PAM recognition through its interaction with the PI domain while simultaneously preventing Cas9 activation by preventing required conformational changes. The structure further suggests that AcrIIA26 binding requires sgRNA, as the Cas9 conformation observed in our structure only forms once the sgRNA is loaded [17].

The 5-helix bundle of AcrIIA26 occupies the same position as the PAM duplex in target-bound Cas9 structures (Figure 5B). The positioning of AcrIIA26 residues E49 and D50 to interact with the critical PAM-recognition residues R1333 and R1335 directly prevents SpyCas9 from recognizing its NGG-PAM sequence (Figure 5C). Our mutagenesis data strongly support this mechanism, as the E49A/D50A double mutant almost completely abolished AcrIIA26 inhibition (Figure 5D). Comparison with other structurally characterized Acr proteins reveals that direct blocking of PAM recognition is a convergent evolutionary strategy. AcrIIA2, AcrIIA4, AcrIIA13, AcrIIA15, and AcrIIC5 all target the PAM-binding pocket despite having distinct protein folds (Figure 6 and Supplementary Figure S4). PAM recognition is the first step in DNA target binding, thus an effective means of inhibiting Cas9 activity. Indeed, the fact that phages have independently evolved multiple structurally unrelated solutions to block this site underscores the effectiveness of this inhibition strategy in the evolutionary arms race between phages and bacteria.

Beyond blocking PAM recognition, AcrIIA26 employs a second inhibition mechanism through its 4-helix bundle, which interacts with the REC1 and REC2 domains in the REC lobe (Figure 4B). Critically, this bundle occupies the same space that the HNH nuclease domain must access during Cas9 activation. In the catalytically active state, the HNH domain undergoes a conformational change to position itself for target strand cleavage. By holding the REC2 domain in a position that would clash with the activated HNH domain, AcrIIA26 effectively locks Cas9 in an inactive conformation (Figure 4E). The complete loss of inhibition observed with the F121A mutant demonstrates that this interaction is essential for AcrIIA26 function (Figure 5D). Thus, preventing Cas9 activation and PAM-blocking are both necessary for AcrIIA26 to inhibit SpyCas9.

All structurally characterized AcrII proteins function, at least in part, by stabilizing inactive Cas9 conformations. AcrIIA26, along with AcrIIA2, AcrIIA4, AcrIIA13, AcrIIA15, and AcrIIC5, binds at the Cas9 DNA-binding site (Figure 6) and additionally prevents HNH domain conformational changes required for DNA cleavage. In contrast, AcrIIA6, AcrIIA25.1, AcrIIA32, and AcrIIC4 bind away from the Cas9 DNA-binding site and stabilize inactive states through allosteric mechanisms that block the conformational changes necessary for DNA binding (Figure 6). AcrIIA14 and AcrIIC3 inhibit Cas9 by preventing HNH conformational changes required for DNA cleavage (Figure 6). This diversity of conformational trapping strategies underscores the importance of multiple conformational checkpoints in the Cas9 catalytic mechanism.

The diverse strategies employed by AcrII proteins to inhibit Cas9 suggest differential capabilities for reducing Cas9 off-target effects in genome editing applications. AcrIIA26 and other Acrs that block PAM recognition prevent initial DNA engagement, potentially eliminating off-target binding at sites with degenerate PAM sequences or partial complementarity. In contrast, Acrs that prevent DNA cleavage only after target binding may still permit Cas9 to occupy off-target sites, potentially interfering with transcription or chromatin structure even without generating double-strand breaks. Furthermore, Acrs that require sgRNA-loaded Cas9, such as AcrIIA26, ensure selective inhibition of catalytically active Cas9, avoiding interference with Cas9 expression and potentially enabling more predictable pharmacokinetics when used as temporal off-switches. The convergent evolution of PAM-blocking mechanisms across structurally unrelated Acrs underscores this vulnerability as a critical control point for therapeutic intervention. However, the mechanistic heterogeneity among REC lobe-binding Acrs indicates that comparative studies will be essential for rational selection of optimal Acr-based control systems. Such studies should evaluate relative efficacies in reducing off-target effects across different genomic contexts, PAM sequences, and mismatch profiles.

In conclusion, the structural and biochemical characterization of the SpyCas9–AcrIIA26 complex advances our understanding of CRISPR inhibition and reveals how phages have exploited fundamental mechanistic requirements of Cas9 function to evade bacterial adaptive immunity. The modular architecture and mechanistic insights provided by our structure offer opportunities for rational engineering of improved Cas9 inhibitors for research and therapeutic applications. Future studies may explore how the unique structural features of AcrIIA26 can be leveraged to improve CRISPR–Cas9 gene editing technologies and for broader biotechnological applications.

## Materials and methods

### Cloning and oligonucleotides

The gene encoding AcrIIA26 (Supplementary Table S1) with an N-His6-TEV tag was cloned into pET28a by Twist Biosciences. AcrIIA26 mutants were generated by inverse PCR followed by blunt end ligation. Primers for the mutagenesis are shown in Supplementary Table S2. Plasmid cloning and maintenance were performed in DH10B cells (Invitrogen). Plasmids were prepared from overnight cultures using the GeneJET Plasmid Miniprep Kit (Thermo Fisher Scientific). All constructs were sequenced and confirmed by Sanger sequencing (Azenta). The target plasmid containing the TRAC gene (Supplementary Table S1) was cloned into pUCIDT-Kan by Integrated DNA Technologies (IDT). Synthetic sgRNA was also obtained from IDT (Supplementary Table S1). The plasmid for expressing SpyCas9 (pMJ806) was a gift from Jennifer Doudna (Addgene plasmid #39312; http://n2t.net/addgene:39312; RRID:Addgene_39312).

### Protein expression and purification

Plasmids expressing either wild-type AcrIIA26 or AcrIIA26 mutants were transformed into T7 express cells (New England Biolabs, NEB). Cells were grown in LB (lysogeny broth) supplemented with kanamycin at 37°C. At an OD_600_ of 0.8, cultures were induced with 1 mM isopropyl β-d-1-thiogalactopyranoside (IPTG) and grown overnight at 18°C. The cells were harvested and resuspended in lysis buffer (50 mM Tris–HCl, pH 8.0, 10 mM imidazole, 0.5 mM TCEP, and 500 mM NaCl) with 1 mM of each protease inhibitor (PMSF, Pepstatin A, Bestatin, and E64). The cells are lysed in an Emulsiflex-C5 homogenizer (Avestin) and centrifuged at 18,000 rpm for 30 min at 4°C. The supernatant is stirred with nickel-charged immobilized metal ion affinity chromatography (IMAC) resin (Bio-Rad) for 1 h at 4°C. The resin was washed with lysis buffer and then bound protein was eluted with elution buffer (50 mM Tris–HCl, pH 8, 500 mM imidazole, 0.5 mM TCEP, and 500 mM NaCl). The protein was then run over a Superdex 75 size-exclusion chromatography column in gel filtration buffer (20 mM HEPES–KOH, pH 7.5, 300 mM NaCl, 1 mM DTT). Fractions were concentrated with a 3k spin column (Millipore), aliquoted, and snap-frozen with liquid nitrogen to store at −80°C. SDS–PAGE analysis of all purified AcrIIA26 proteins is shown in Supplementary Figure S5A.

The SpyCas9 expression plasmid was transformed into BL21 Rosetta (DE3) cells (EMD Biosciences). Cells were grown in Terrific Broth supplemented with kanamycin at 37**°**C. At an OD_600_ of 0.6, cultures were induced with 1 mM IPTG and grown overnight at 18**°**C. The cells were harvested and resuspended in lysis buffer (50 mM Tris–HCl, pH 8.0, 10 mM imidazole, 0.5 mM TCEP, 5% (v/v) glycerol, and 500 mM NaCl) with 1 mM of each protease inhibitor (PMSF, Pepstatin A, Bestatin, and E64). The cells are lysed in an Emulsiflex-C5 homogenizer (Avestin) and centrifuged at 18,000 rpm for 30 min at 4**°**C. The supernatant is stirred with nickel-charged IMAC resin (Bio-Rad) for 1 h at 4**°**C. The resin was washed with lysis buffer, and then bound protein was eluted with elution buffer (50 mM Tris–HCl, pH 8, 300 mM imidazole, 0.5 mM TCEP, 5% (v/v) glycerol, and 500 mM NaCl). The his-MBP tag was removed by incubation with TEV protease at 4**°**C for 1 h. The resulting mixture was passed over Nickel IMAC resin for a second time. The flow-through was further purified using a HiLoad 26/600 S200 Superdex column (GE Healthcare) equilibrated in 20 mM HEPES–NaOH pH 7.3, 100 mM KCl, and 5 mM DTT. Fractions were concentrated with a 30k spin column (Millipore), aliquoted, and snap-frozen with liquid nitrogen to store at −80**°**C. SDS–PAGE analysis of all purified Cas9 is shown in Supplementary Figure S5B.

### DNA cleavage assays

The sgRNA was annealed at 94**°**C for 2 min and incubated on ice for 20 min. Cas9 proteins (350 nM) and sgRNA (350 nM) were mixed in Cas9 gel filtration buffer and incubated at 37**°**C for 10 min. The Cas9–sgRNA complex was then diluted into NEB’s rCutSmart^™^ buffer (50 mM potassium acetate, 20 mM Tris–acetate, pH 7.9, 10 mM magnesium acetate, and 100 μg/ml recombinant albumin) before the addition of the indicated concentration of AcrIIA26. Reactions were then incubated at room temperature for 20 min. Reactions were started by the addition of 4 nM of target plasmid and incubated for 10 min at 37**°**C. The reaction was quenched by adding an equal volume of phenol:chloroform:isoamyl alcohol and extracted. Final samples were analyzed on 0.8% agarose gels post-stained with ethidium bromide. At least three independent replicates were collected for all assays.

### Cryo-EM sample preparation and data collection

To prepare the sample, the sgRNA was annealed at 94**°**C for 2 min and incubated on ice for 20 min. Cas9 proteins (4 μM) and sgRNA (4 μM) were mixed in buffer (resulting in 1 mM magnesium acetate, 20 mM HEPES, pH 7.5, 100 mM KCl, 25 mM NaCl, and 5 mM DTT) and incubated at 37**°**C for 10 min. The Cas9–sgRNA complex was then incubated with 8 μM AcrIIA26 for 20 min at room temperature.

Holey carbon copper grids with a graphene oxide support film (EMS, GOHC300CU) were glow discharged for 7 s at 15 mA using a PELCO easiGLow™. Four microliters of the Cas9–sgRNA–AcrIIA26 complex were applied to the grid at 7**°**C in 100% relative humidity for 20 s, followed by blotting for 1.5 s and vitrification into liquid ethane using a Vitrobot Mark IV (Thermo Fisher Scientific, U.S.A.) and stored in liquid nitrogen. Data were collected using a Thermo Fisher G3i Titan Krios equipped with a Falcon 4i camera and Selectris energy filter (Beckman Center for CryoEM at Johns Hopkins University) operated at 300 keV with a nominal magnification of 130,000× (calibrated pixel size of 0.93 Å). A total of 8708 movies were collected using Thermo Fisher E Pluribus Unum in counting mode and recorded in electron event representation (EER) format, with a defocus range of −0.5 to −3.0 μm. Each movie was recorded with a nominal total dose of 40 e^−^ Å^−2^. EER movies were converted to tiff format using an EER grouping of 40 frames to give a dose fractionation of approximately 1 e^−^ Å^−2^.

### Cryo-EM data processing

The data processing pipeline is summarized in Supplementary Figure S1. All processing steps were performed with default options unless otherwise stated. Movies were gain-corrected, aligned, and dose-weighted using RELION-5’s [55] implementation of MotionCor2 [56] with an EER fractionization of 40. The contrast transfer function (CTF) parameters were estimated using CTFFIND-4.1 [57]. A total of 698,543 particles were automatically picked with crYOLO [58] using the default model and a box size of 150 pixels. Particle coordinates were imported into RELION and extracted with a box size of 256 pixels without down-sampling. Particle images were imported into cryoSPARC v4.6 [58,59]. All particles were then subjected to *ab initio* reconstruction and heterogeneous refinement asking for three classes. This resulted in one good Cas9 class (302,159 particles) and two junk classes. The good class was nonuniform, refined, and subjected to 3D classification at 6 Å resolution, asking for ten classes and forcing hard classification. One of the resulting classes, which showed continuous and clear density for Cas9, AcrIIA26, and sgRNA (59,914 particles), was nonuniform refined while optimizing per-particle defocus and per-group CTF parameters. This resulted in a map at 2.98 Å resolution (Supplementary Figures S1 and S2). However, density describing the HNH domain was much lower resolution than the rest of the complex, 4.14 Å (Supplementary Figure S1). Therefore, particle subtraction was performed to isolate the HNH domain. The subtracted particles were then subjected to local refinement using a rotation and shift search extent of 1.5° and 0.5 Å, respectively. This improved the resolution of the HNH domain to 3.27 Å (Supplementary Figures S1 and S2). We generated a composite map by combining the two maps with the volume maximum command in UCSF ChimeraX (v1.4) [60]. Map sharpening was performed with DeepEMhancer [61]. Cryo-EM data collection, refinement, and validation statistics are presented in Supplementary Table S3.

### Model building and refinement

The structure of the Cas9–sgRNA–AcrIIA26 complex was predicted with AlphaFold3 [62]. The model was segmented into domains, which were then individually rigid-body fitted into the combined map using ChimeraX (v1.4). The resulting model was refined with ISOLDE (v1.4b2). A final round of real-space refinement of the model was performed in Phenix (v1.20.1). All refinement steps were carried out with the unsharpened combined map.

## Supplementary Material

Supplementary Figures S1-S5 and Tables S1-S3

## Data Availability

The cryo-EM density maps have been deposited in the Worldwide Protein Data Bank under the accession code EMD-74542 [63] and the coordinates have been deposited in the Protein Data Bank (PDB) under the accession code 9ZQ6 [64]. All other data are included within the main article and its supplemental files. Experimental materials are available from the corresponding author on request.

## Open Access

Open access for this article was enabled by the participation of Johns Hopkins University in an all-inclusive *Read & Publish* agreement with Portland Press and the Biochemical Society.

## Abbreviations

4HB: 4-helix bundle
5HB: 5-helix bundle
Acr: anti-CRISPRs
Cas: CRISPR-associated
CRISPR: clustered regularly interspaced short palindromic repeats
crRNA: CRISPR RNA
Cryo-EM: cryogenic electron microscopy
DSB: double-stranded break
PAM: protospacer adjacent motif
r.m.s.d.: root-mean-square deviation
sgRNA: single-guide RNA
tracrRNA: *trans*-activating crRNA
