# Supplementary material for: Structural basis for inhibition of SpyCas9 by the anti-CRISPR protein AcrIIA26
Source: Biochem J. 2026 Feb 6;483(3):289–300. doi: 10.1042/BCJ20250364 (PMC13089141; doi:10.1042/BCJ20250364)
Supplement: Supplementary Figures S1-S5 and Tables S1-S3 [file BCJ-2025-0364_supp.pdf]

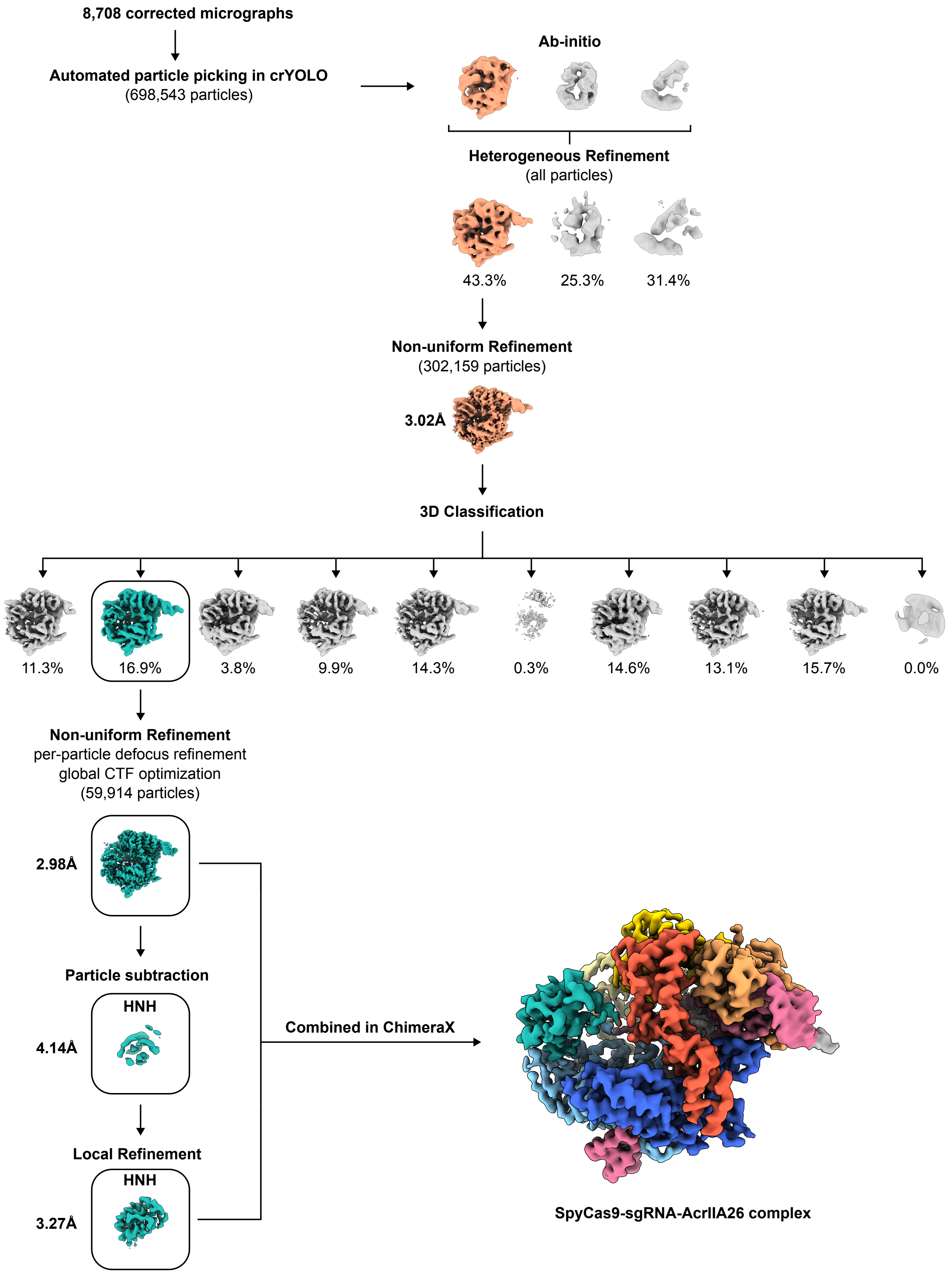

Figure S1. Cryo-EM data processing workflow

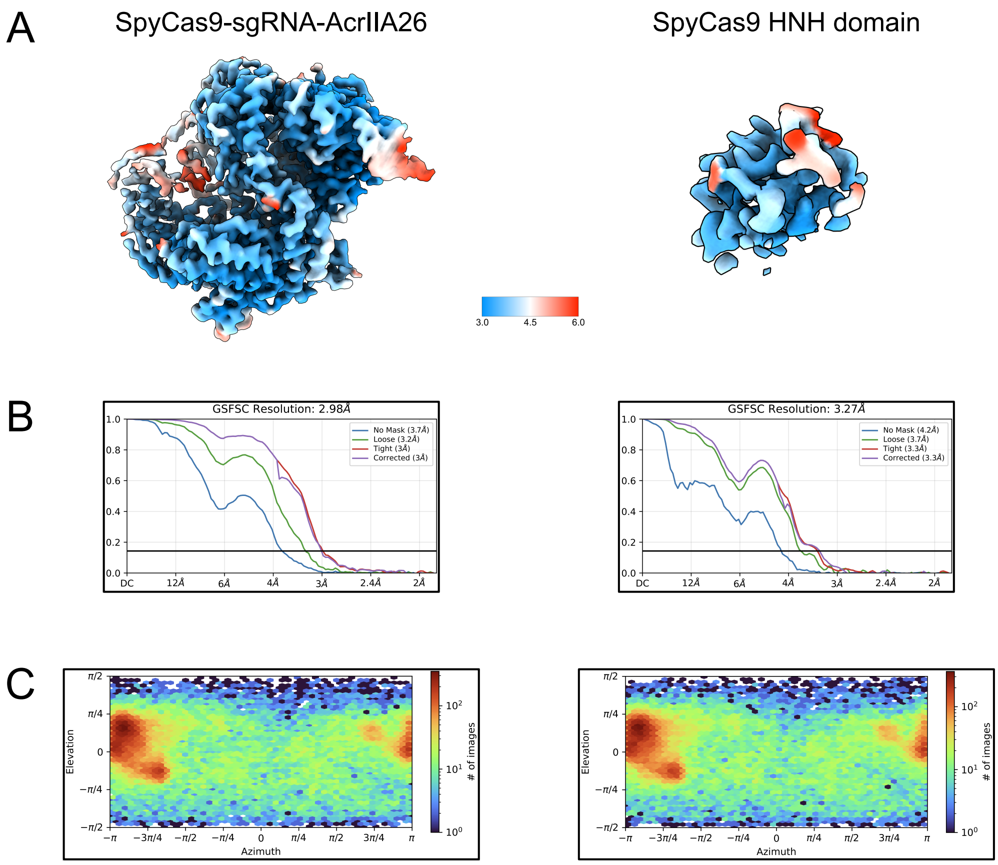

Figure S2. Resolution estimates and orientation distributions of cryo-EM maps

(A) Unsharpened maps colored according to local resolution
(B) Gold-standard FSC curves for cryo-EM reconstructions (FSC = 0.143)
(C) Euler diagrams showing orientation distributions of cryo-EM reconstructions

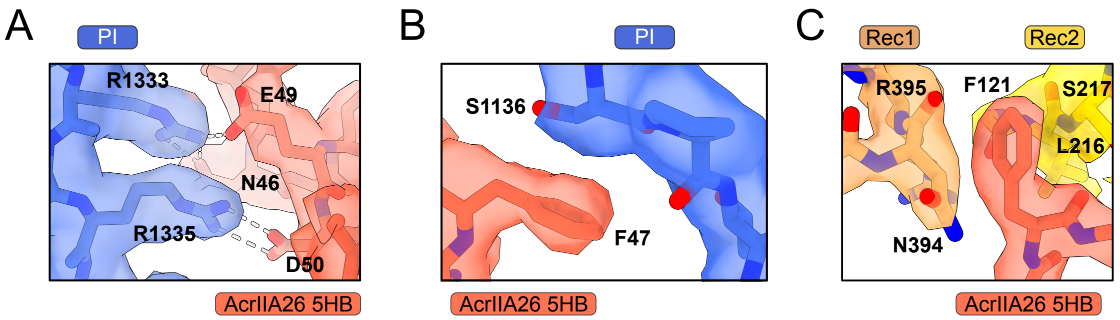

Figure S3 Close-up of density at AcrIIA26 - Cas9 contact points

(A) Density describing how AcrIIA26 interacts with the PAM recognition residues from SpyCas9
(B) Density describing of AcrIIA26 F47 interacts with S1136 of Spy Cas9
(C) Density describing how AcrIIA26 F121 interacts with Spy Cas9

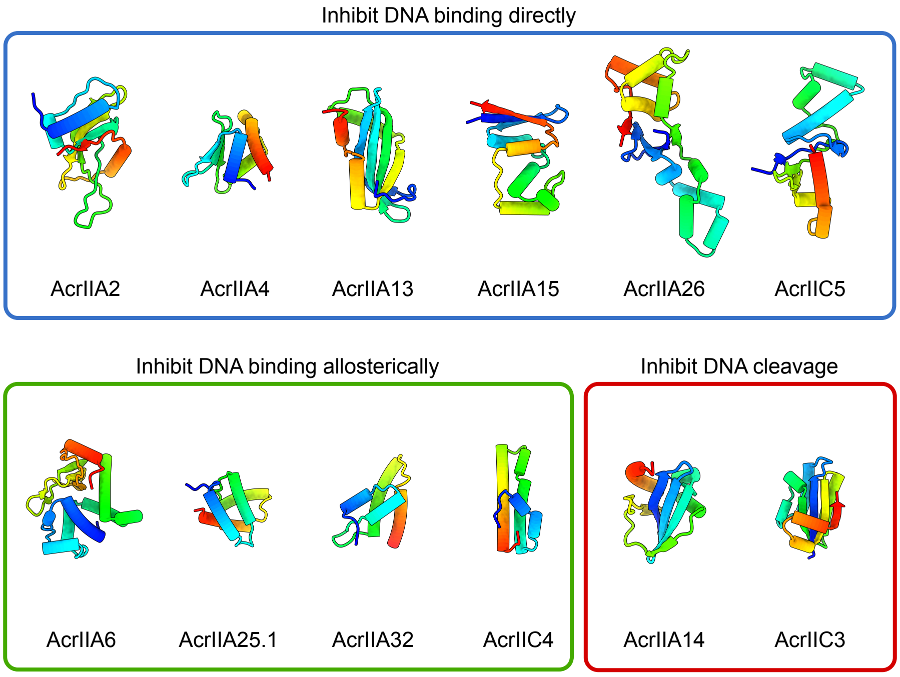

Figure S4: Structures of AcrII proteins

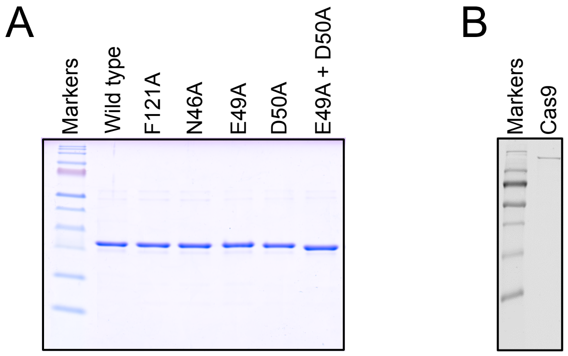

Figure S5: Purification of AcrIIA26 and SpyCas9

(A) SDS-PAGE of wild-type and mutant AcrIIA26.
(B) SDS-PAGE of Spy Cas9

Table S1. Sequences of DNA and RNA reagents used in this study

Name	Sequence (5’ to 3’)
AcrIIA26	ATGGGCAGCAGCCATCATCATCATCATCACAGCAGCGGCGAGAATCTTTA<br>TTTTCAGGGCCATATGAAAAAACTTTATATCCAAACAAACCAATTCGCTA<br>ACGGTGAGCTTCAAGTTGAAAATACTAGCTATGAACTTTGTGATACTTTC<br>AAAGAGCTATACTCAGTCGCTTCAAATTTGGTTGATGAAAACACACTGAA<br>TTTCGTTGAGGATAATTTCATTGAACAAAATTACAAAGATGAATACAATG<br>GTGTTTACGAAAACGATGGAGATACAGGCGAATTTGTAGGGCAAGTCTTT<br>GAAAACAAAGTCACTGAAGAACAGTTCAAAGAATTACTTGAACAACTTGA<br>AATCACTTATACTGAATTTGATCCAGAAGAAGAACTTGCAAAATGTATCG<br>CAAATAAAAACCGTAAATCTGAATTTTACGGTAACGGATTAAAAGTTATC<br>GCTGAATATCTTGAAAGCATTTCTCACGAAGATGCGCTCGCAGTTGTTAC<br>TTATTACTATTTCTATTTCGGATTCGGTTACGAAGACCAACTTATCTCAG<br>ATATTAAAGATGATCAAGAAGATGGTGTGAAATTTGAACACGTTGAACGG<br>TCTGAAACCATCTAA
TRAC	ATGGCATGCCCTGGCTTCCTGTGGGCACTTGTGATCTCCACCTGTCTTGA<br>ATTTAGCATGGCTCAGACAGTCACTCAGTCTCAACCAGAGATGTCTGTGC<br>AGGAGGCAGAGACCGTGACCCTGAGCTGCACATATGACACCAGTGAGAGT<br>GATTATTATTTATTCTGGTACAAGCAGCCTCCCAGCAGGCAGATGATTCT<br>CGTTATTCGCCAAGAAGCTTATAAGCAACAGAATGCAACAGAGAATCGTT<br>TCTCTGTGAACTTCCAGAAAGCAGCCAAATCCTTCAGTCTCAAGATCTCA<br>GACTCACAGCTGGGGGATGCCGCGATGTATTTCTGTGCTTATAGGAGCGC<br>GGTAAACGCGAGACTCATGTTTGGAGATGGAACTCAGCTGGTGGTGAAGC<br>CCAATATCCAGAACCCTGACCCTGCCGTGTACCAGCTGAGAGACTCTAAA<br>TCCAGTGACAAGTCTGTCTGCCTATTCACCGATTTTGATTCTCAAACAAA<br>TGTGTCACAAAGTAAGGATTCTGATGTGTATATCACAGACAAAACTGTGC<br>TAGACATGAGGTCTATGGACTTCAAGAGCAACAGTGCTGTGGCCTGGAGC<br>AACAAATCTGACTTTGCATGTGCAAACGCCTTCAACAACAGCATTATTCC<br>AGAAGACACCTTCTTCCCCAGCCCAGAAAGTTCCTGTGATGTCAAGCTGG<br>TCGAGAAAAGCTTTGAAACAGATACGAACCTAAACTTTCAAAACCTGTCA<br>GTGATTGGGTTCCGAATCCTCCTCCTGAAAGTGGCCGGGTTTAATCTGCT<br>CATGACGCTGCGGCTGTGGTCCAGC
sgRNA	GGAGAGUCUCUCAGCUGGUACAGUUUUAGAGCUAUGCUGUUUUGAAAAAA<br>ACAGCAUAGCAAGUUAAAAUAAGGCUAGUCCGUUAUCAACUUGAAAAAGU<br>GGCACCGAGUCGGUGCUUCG

Table S2. Sequences of DNA primers used in this study

Name	Sequence (5’ to 3’)
N46A-F	GCTTTCGTTGAGGATAATTTCATTGAAC
N46A-R	CAGTGTGTTTTCATCAACCAAATTTG
E49A-F	GCTGATAATTTCATTGAACAAAATTAC
E49A-R	AACGAAATTCAGTGTGTTTTCATC
D50A-F	GCTAATTTCATTGAACAAAATTACAAAG
D50A-R	CTCAACGAAATTCAGTGTGTTTTCATC
E49A+D50A-F	GCTAATTTCATTGAACAAAATTACAAAG
E49A+D50A-R	AGCAACGAAATTCAGTGTGTTTTCATC
F121A-F	GCTTACGGTAACGGATTAAAAGTTATCG
F121A-R	TTCAGATTTACGGTTTTTATTTGCGATAC

Table S3. Cryo-EM data collection, refinement and validation statistics

**Data Collection and processing**	
Microscope	ThermoFisher G3i Titan Krios
Camera	Falcon 4i
Energy filter	Selectris
Magnification	130,000
Voltage (kV)	300
Electron fluence ($e^-/Å^2$)	40
Electron fluence per frame ($e^-/Å^2/\mathrm{frame}$)	1
Number of frames per movie	40
Number of movies used	8708
Defocus range (μM)	-0.5 to 3.0
Pixel Size (Å)	0.93
Symmetry imposed	C1
Initial number of particle images	698,543
Final number of particle images	59,914
Global Map resolution (Å) (FSC=0.143)	3.0
Particle box size (pixels)	256
**Refinement**	
Initial model used	AF3 model
Model Resolution (Å) (FSC=0.5)	3.26
Map Sharpening B factor ($Å^2$)	72
Protein residues	1,476
Nucleotides	83
r.m.s.d	
Bond lengths (Å)	0.004
Bond angles (°)	1.000
**Validation**	
Clashscore	1.63
MolProbity Score	1.18
Poor rotamers (%)	0.08
Ramachandran plot:	
Favored (%)	96.07
Allowed (%)	3.93
Disallowed (%)	0
